# MiR-193b, downregulated in Ewing Sarcoma, targets the ErbB4 oncogene to inhibit anchorage-independent growth

**DOI:** 10.1371/journal.pone.0178028

**Published:** 2017-05-18

**Authors:** Colin Moore, Janet K. Parrish, Paul Jedlicka

**Affiliations:** 1 Center for Cancer and Blood Disorders, Children’s Hospital Colorado, Aurora, Colorado, United States of America; 2 Department of Pathology, University of Colorado Denver, Anschutz Medical Campus, Aurora, Colorado, United States of America; Johns Hopkins University, UNITED STATES

## Abstract

Ewing Sarcoma is an aggressive, oncofusion-driven, malignant neoplasm of bone and soft tissue affecting predominantly children and young adults. Seeking to identify potential novel therapeutic targets/agents for this disease, our previous studies uncovered microRNAs regulated by EWS/Fli1, the most common oncofusion, with growth modulatory properties. In the present study, we sought to identify EWS/Fli1-repressed, growth suppressive, microRNAs potentially amenable to replacement in Ewing Sarcoma cells. Eight microRNAs (143, 153, 184, 193b, 195, 203, 206 and 223) were selected for evaluation as EWS/Fli1-repressed and underexpressed in Ewing Sarcoma cells, and reported to be growth suppressive in other pediatric or/and adult cancers. The selected miRs, and appropriate non-targeting controls, were introduced into two different Ewing Sarcoma cell lines (A673 and SK-ES-1), and effects on growth were examined using a high and low-density growth assay. MiR-193b was growth inhibitory in both assays and cell lines. In subsequent analyses, we found that stable overexpression of miR-193b also inhibits anchorage-independent growth in both A673 and SK-ES-1 cells. We further show that miR-193b negatively regulates expression of the ErbB4 oncogene in A673 and SK-ES-1 cells, and that depletion of ErbB4 is itself inhibitory to anchorage-independent growth in the same cell lines. Together, our studies show that the EWS/Fli1-repressed miR-193b is growth suppressive in Ewing Sarcoma, and identify ErbB4 as a target gene and candidate mediator of this growth suppression.

## Introduction

Ewing Sarcoma is a cancer of bone and soft tissue predominantly affecting the pediatric age group [1]. It is an aggressive malignancy with frequently poor outcomes, especially in patients presenting with metastatic disease or relapse [1]. The pathogenesis of Ewing Sarcoma is driven by EWS/Ets fusion oncoproteins, which arise as a consequence of recurrent chromosomal translocations [2]. EWS/Ets oncofusions, of which EWS/Fli1 is by far the most common, are aberrant transcriptional and post-transcriptional regulators, which enact widespread dysregulation of the expressed genome, including protein-coding genes, microRNAs and other non-coding RNAs [2–4].

MicroRNAs (miRs) play an important role in the regulation of gene expression in normal physiology and disease [5]. MiRs are short (20–30 nucleotide) RNA molecules that bind to protein-coding messenger RNA (mRNA), predominantly in the 3’ untranslated region (UTR) [5]. This binding results in decreased gene expression, by mechanisms including increased mRNA degradation and inhibition of translation [5]. MiR/mRNA interactions are sequence-specific, but involve a limited (~6–8) nucleotide match [6]. Thus, individual miRs have many possible mRNA targets, while, as a group, miRs contribute to the control of expression of the majority of the genome. In cancer, miRs function as context-dependent tumor suppressors or oncogenes, capable, through their molecular function as regulators of gene expression, of modifying all aspects of tumorigenesis, including tumor cell proliferation, apoptosis, invasion/metastasis, stem-like properties and angiogenesis [7, 8]. MiRs also represent potential therapeutic agents or/and targets in cancer [9–13].

Seeking to identify new EWS/Ets-driven oncogenic pathways with targeting potential in Ewing Sarcoma, our laboratory previously performed global miR profiling upon silencing of the EWS/Fli1 oncoprotein [14]. This study identified miRs subject to positive and negative regulation by EWS/Fli1 [14], representing candidate pro-oncogenic and anti-oncogenic miRs, respectively. In follow-up functional studies, we validated oncogenesis-modifying roles for several individual EWS/Fli1-regulated miRs or/and miR clusters in Ewing Sarcoma [14–16]. The goal of the present study was to identify and characterize additional anti-oncogenic miR(s) amenable to potential therapeutic replacement in this disease.

## Materials and methods

### Cell culture

The origin and culture of the Ewing Sarcoma cell lines A673 and SK-ES-1, and human Mesenchymal Stem Cells (hMSC; Lonza) have been previously described [14]. The Ewing Sarcoma cell lines were authenticated at our institution by STR profiling, and all cell lines were repeatedly verified to be mycoplasma-free.

### Ectopic microRNA expression

Transient ectopic microRNA (miR) expression was achieved using transfection of miR mimics, or non-targeting negative control mimic (25 nM; Applied Biosystems, Carlsbad, CA, USA), using Lipofectamine 2000, as previously described [14]. For stable ectopic expression of miR-193b, the miR-193b genomic locus, including approximately 250 bp of upstream and downstream flanking sequence, was cloned from Ewing Sarcoma A673 cells into the pMSCV-Puro retroviral expression vector, using standard molecular techniques and verification by sequencing. Replication-incompetent, VSV-G pseudotyped, infectious retrovirus was prepared as previously described [14], using a retroviral packaging system. Transduction of Ewing Sarcoma cells with viral supernatant, and subsequent selection with Puromycin (2 μg/ml), was performed as previously described [14]. Control cells were infected with empty pMSCV-Puro.

### Gene expression silencing

shRNA-mediated gene expression silencing via lentiviral delivery was performed as previously described [17]. The control, non-targeting shRNA consisted of a scrambled sequence (Addgene plasmid 1864; [18]). Specific shRNAs targeting ErbB4 were obtained from Sigma (Mission shRNA, distributed via the University of Colorado Cancer Center Functional Genomics Core Facility). ShRNAs 1 and 2 for ErbB4 correspond to TRCN0000001411 and TRCN0000379973, respectively.

### Growth assays

For MTT assays, one day following miR transfection, cells were counted and replated at a density of 5,000 cells/well in 96-well plates. On subsequent days 1, 3 and 5, cell growth was measured using an MTT assay, as described [19]. For clonogenic assays, the same miR-transfected cells were plated at a density of 500 cells/well in 6-well plates. After 14 days, the cells were washed with PBS and then stained with 0.1% crystal violet in 25% methanol. Colonies were quantified using NIS-Elements System Software, as previously described [16]. For anchorage-independent colony growth in soft agar, 10,000 cells/well in 6-well plates were grown in 0.4% agar (Difco Agar Noble (BD 214230)) and growth medium containing 20% FBS. Colonies were stained with Nitroblue Tetrazolium Chloride, and quantified using the NIS-Elements System Software, as previously described [14].

### MicroRNA expression level analysis

Quantification of miR-193b levels in Ewing Sarcoma cell lines and MSCs was performed using RT-qPCR, as previously described [14]. Primers were obtained from Qiagen.

### Western blotting

Protein extract preparation, SDS-PAGE, immunoblotting and detection were performed as previously described [16]. The primary antibody for ErbB4 detection was obtained from Cell Signaling Technologies (#4795), and was used at 1:1000 dilution.

### Luciferase assays

For 3’UTR activity assays, Ewing Sarcoma cells were transiently cotransfected using Lipofectamine 2000 with a psiCHECK-2 reporter construct containing the wild-type ErbB4 3’UTR miR-193b site or the mutated site (introduced via standard cloning techniques followed by sequence verification), and miR-193b mimic, non-targeting negative control mimic, or no nucleic acid (Mock group). Reporter activity was determined 48 hours later, as described previously [14].

## Results

### MiR-193b is a new growth-suppressive microRNA in Ewing Sarcoma

Using global microRNA (miR) profiling, our laboratory previously identified miRs with altered expression as a function of EWS/Fli1 levels in Ewing Sarcoma, including miRs that were upregulated and others that were downregulated by the EWS/Fli1 oncoprotein [14]. We hypothesized that EWS/Fli1-induced miRs would include candidate pro-oncogenic miRs, while EWS/Fli1-repressed miRs would include candidate anti-oncogenic miRs in Ewing Sarcoma. Our own functional analyses of several selected miRs provided support for this hypothesis, identifying both EWS/Fli1-repressed growth suppressive miRs and EWS/Fli1-induced growth-promotional miRs [14–16]. We were particularly intrigued by EWS/Fli1-repressed, anti-oncogenic miRs, as such miRs could conceivably be introduced into Ewing Sarcoma cells to inhibit cancer phenotypes. The goal of the present study, initially undertaken in parallel to our other functional and mechanistic analyses [14–16], was to identify additional candidates for miR replacement in Ewing Sarcoma. We began with miRs identified as EWS/Fli1-repressed in our original expression screen [14], using a false discovery rate (FDR) cutoff of <0.05. From this group, we selected miRs that met two or more of the following three criteria: 1) miRs shown to have predominantly or exclusively anti-oncogenic effects in other cancers; 2) miRs with target profiles relevant to Ewing Sarcoma oncogenesis; and 3) miRs expressed at relatively low levels in Ewing Sarcoma cells (and thus potentially more amenable to replacement). Using these criteria, eight EWS/Fli1-repressed miRs were selected for further analysis.

**Fig 1:**
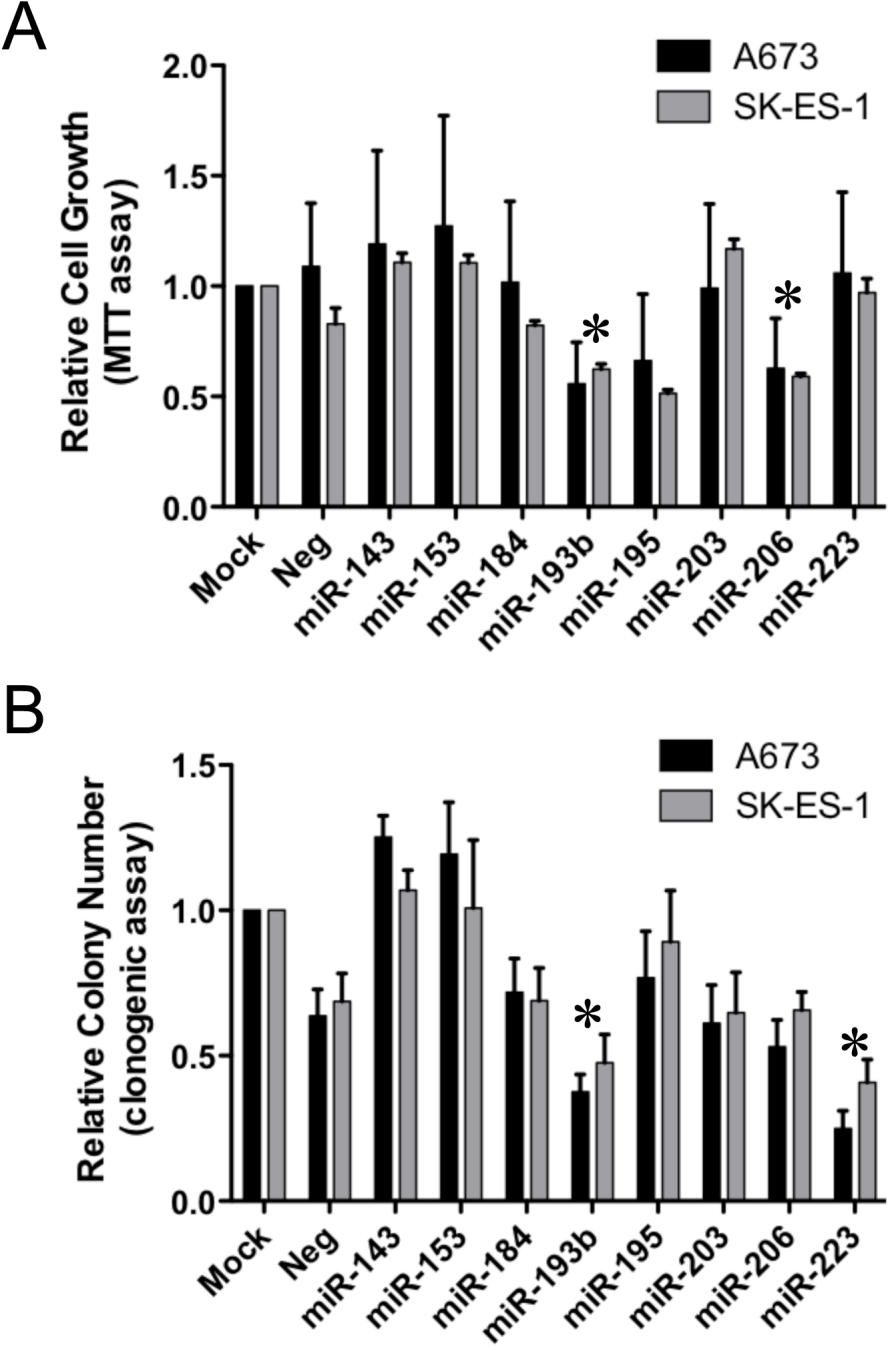
Functional miniscreen for growth suppressive microRNAs in Ewing Sarcoma. (A) Ewing sarcoma A673 and SK-ES-1 cells were mock transfected, or transfected with the indicated miR or non-targeting negative control mimics at a final concentration of 25 nM. 24 hours later, cells were harvested, counted and plated at 5 x 10^3^ cells per well in 96-well plates. Relative cell numbers on day 5, normalized to the mock group and plotted as the mean and standard error of the mean (SEM) of 4 independent experiments, each performed in triplicate, are shown. (B) Ewing sarcoma A673 and SK-ES-1 cells were transfected as in “A” and 24 hours later were plated at 500 cells per well in 6-well plates. Colonies were stained with crystal violet 14 days later, and quantified using a Nikon digital image analysis system (NIS-Elements). Results are represented as the mean and SEM of 4 independent experiments, each performed in triplicate, normalized to the mock group. *: p<0.05, in both A673 and SK-ES-1 cells, relative to negative control (“Neg”, which is the average of two different non-targeting negative control mimics).

The selected miRs (143, 153, 184, 193b, 195, 203, 206 and 223) were then subjected to a functional miniscreen using inhibition of growth as the readout. Specifically, miRs were introduced into two different Ewing Sarcoma cell lines (A673 and SK-ES-1) using transient transfection of miR mimics (and corresponding non-targeting negative control mimics), and effects on cell growth were monitored using two different assays: an MTT assay (measuring growth under standard moderate-density culture conditions), and a clonogenic assay (measuring colony initiation and growth under low-density plating conditions). Each assay was repeated four independent times to ascertain reproducibility of effects. The results, plotted as fractional growth relative to mock-transfected control cells, are shown in Fig 1. In the MTT assay, miR-193b and miR-206 consistently inhibited the growth of both A673 and SK-ES-1 cells, while in the clonogenic assay, miR-193b and miR-223 showed the most potent inhibition of colony formation in both cell lines.

### Stable ectopic expression of miR-193b inhibits anchorage-independent growth of Ewing Sarcoma cells

**Fig 2:**
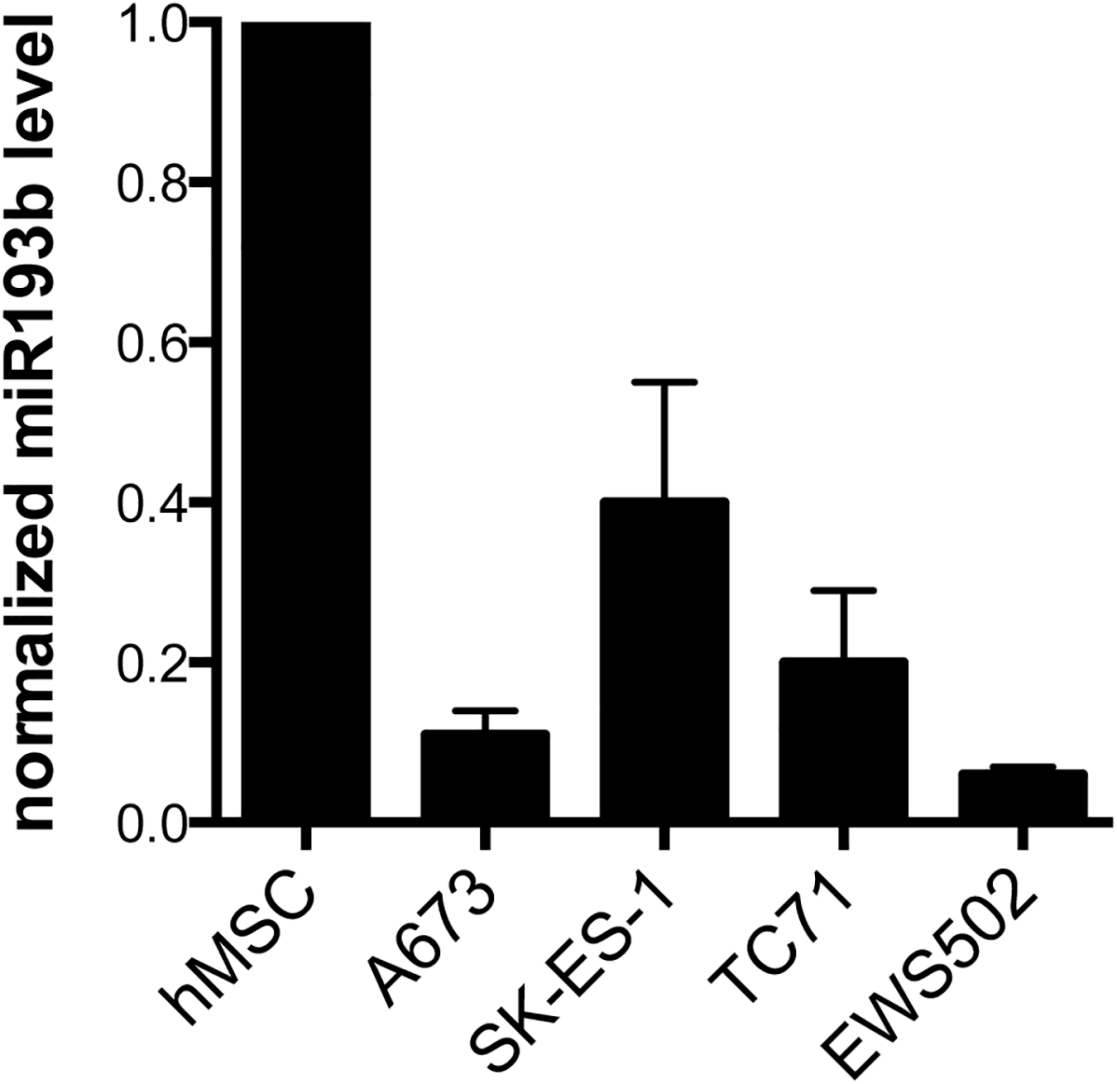
MiR-193b expression. Relative miR-193b levels in human mesenchymal stem cells (hMSC) and four different Ewing Sarcoma cell lines (A673, SK-ES-1, TC71 and EWS502), as measured using RT-qPCR with U6 as the internal control; mean and standard deviation of triplicate samples, normalized to hMSCs.

**Fig 3:**
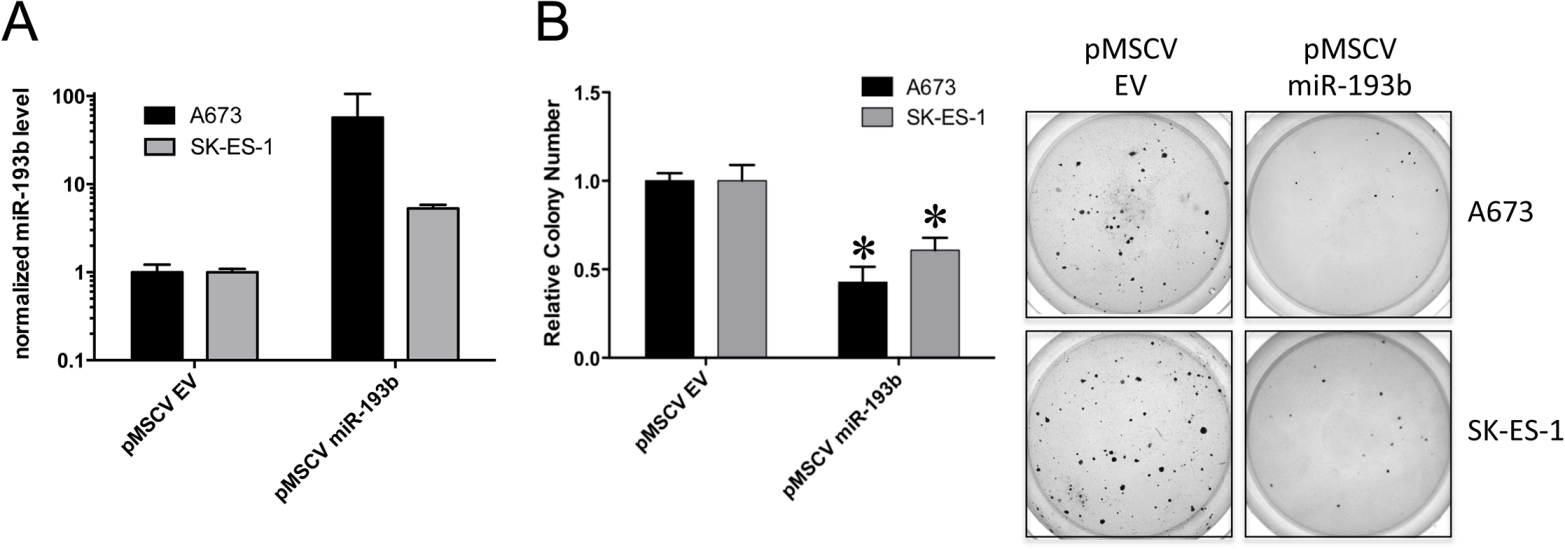
Stable ectopic expression of miR-193b inhibits anchorage-independent growth in soft agar. Ewing sarcoma A673 and SK-ES-1 cells were stably transduced with the pMSCV-Puro retroviral vector expressing miR-193b, or empty vector (EV) control. (A) Relative miR-193b expression levels following selection were determined using RT-qPCR, with U6 as the internal control; mean and standard error of the mean (SEM) of two independent experiments, each performed in triplicate, normalized to empty vector control. (B) For soft agar assays, cells were harvested, counted and plated at 10,000 cells per well in 6-well plates containing 0.4% agar and growth medium with 20% serum. Colonies were stained with nitroblue tetrazolium 2–3 weeks later, and quantified using Nikon digital image analysis system (NIS-Elements). Results are represented as the mean and standard error of the mean (SEM) of 3 independent experiments, each performed in triplicate (*: p<0.05, relative to empty vector control); images from one experiment are also shown.

Given our finding of a growth suppressive phenotype effected by miR-193b in two different cell lines and growth assays, we focused our further studies on this miR. To expand on our observation of miR-193b repression by EWS/Fli1 [14], we compared miR-193b expression levels between Ewing Sarcoma cell lines and human mesenchymal stem cells (hMSC), a putative cell of Ewing Sarcoma origin. As we previously observed for other EWS/Fli1-repressed miRs in our studies [14], miR-193b levels were lower in Ewing Sarcoma cell lines relative to hMSCs (Fig 2), further verifying miR-193b downregulation in Ewing Sarcoma. We next examined the effects of forced, stable, ectopic expression of miR-193b on Ewing Sarcoma growth. MiR-193b was stably expressed in A673 and SK-ES-1 cells using a retroviral system, and growth effects were assessed using anchorage-independent growth in soft agar. As shown in Fig 3, stable ectopic expression of miR-193b robustly inhibited the anchorage-independent growth of both cell lines, compared to controls (cell transduced with empty vector). This finding corroborated our initial observation of the growth suppressive effects of miR-193b using a different expression and assay system, and, moreover, one more closely modeling tumor growth.

### MiR-193b negatively regulates ErbB4 expression in Ewing Sarcoma

**Fig 4:**
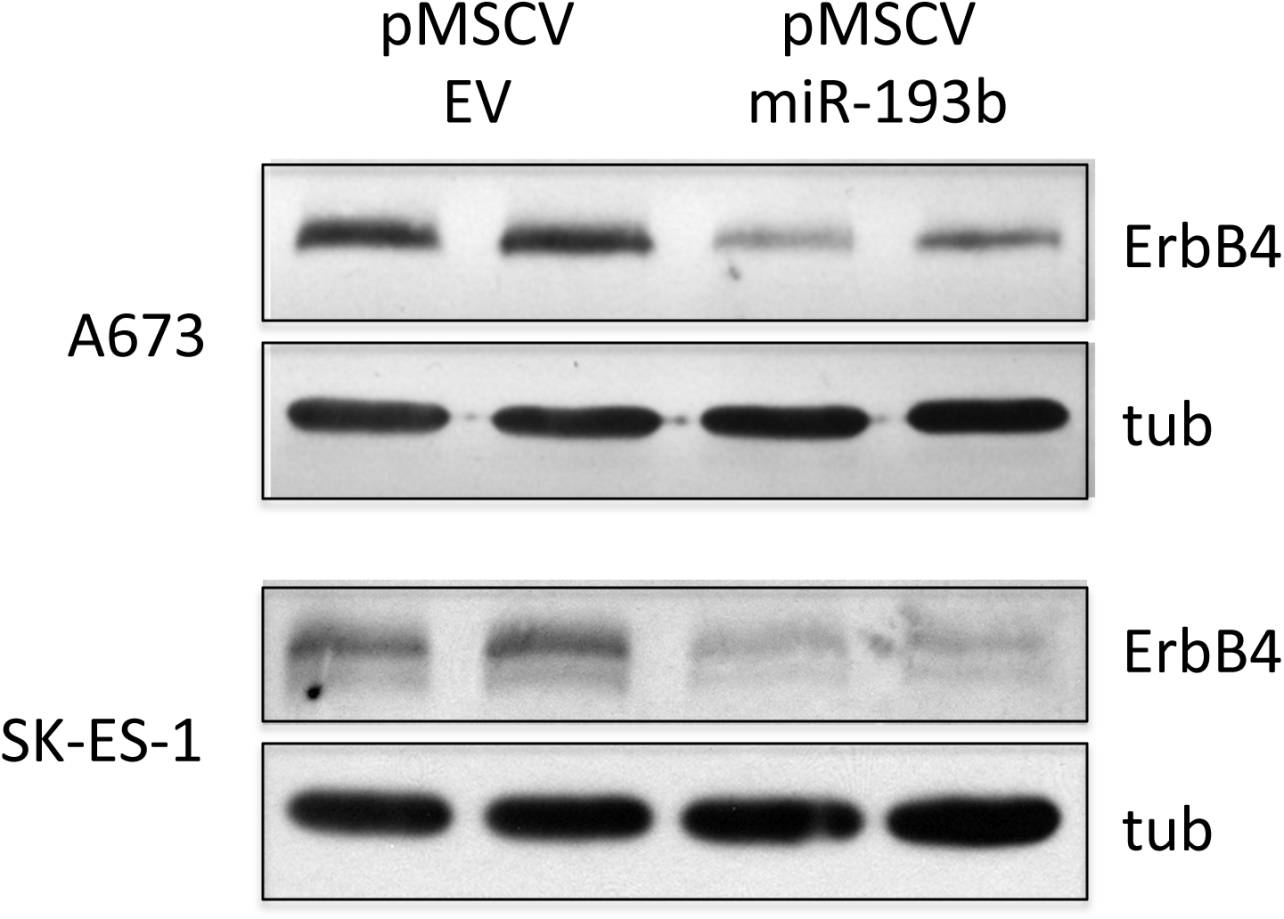
MiR-193b negatively regulates ErbB4 protein expression in Ewing Sarcoma. Ewing sarcoma A673 and SK-ES-1 cells stably expressing empty vector control or miR-193b were harvested for protein extract preparation, and immunoblotting with antibody to ErbB4 and tubulin as loading control.

**Fig 5:**
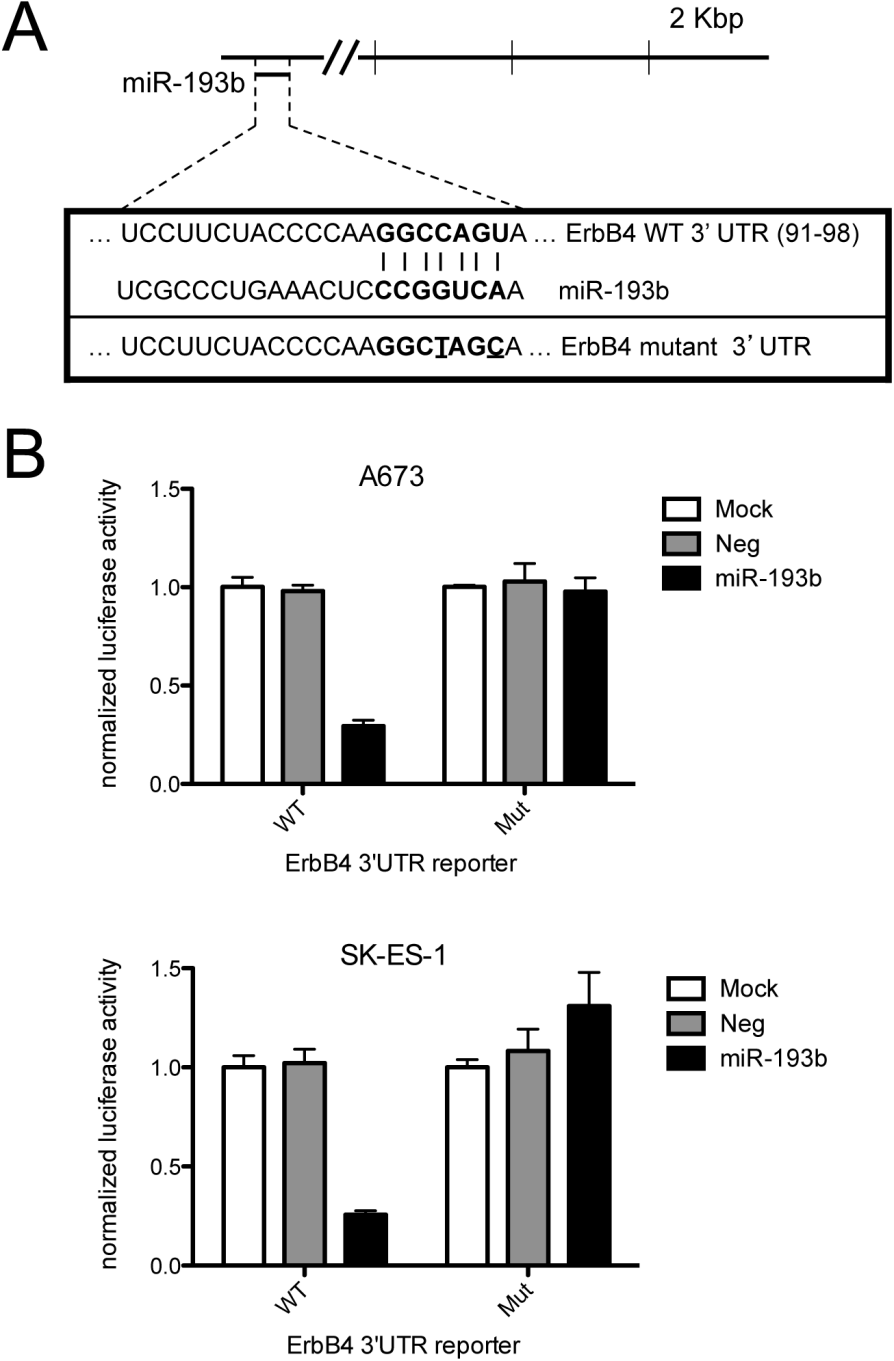
MiR-193b negatively regulates its conserved, predicted binding site in the ErbB4 proximal 3’UTR. (A) Schematic of the ErbB4 3’UTR with location of predicted conserved miR targeting sequences; wild-type and mutant miR-193b target sequences used in reporter assays (the mutated nucleotides are underlined). (B) A673 and SK-ES-1 cells were transfected with a psiCHECK-2 reporter construct containing the wild-type ErbB4 3’UTR miR-193b site or the mutated site, and either mock transfected, or cotransfected with miR-193b mimic or non-targeting negative control mimic. Luciferase reporter activity, normalized to Renilla luciferase activity, was quantified by luminometry; Luciferase/Renilla activity ratio was arbitrarily set to 1 in the mock-transfected group; mean and standard deviation of triplicate transfections.

To gain insight into potential mechanisms of growth inhibition by miR-193b in Ewing Sarcoma, we queried miR target prediction algorithms, including the pathway-focused algorithm DIANA-mirPath. This analysis identified the ErbB4 oncogene as one of the top candidates for regulation by miR-193b. Interestingly, ErbB4 had previously been identified as a suppressor of anoikis in Ewing Sarcoma [20], a phenotype pertinent to anchorage-independent growth. To determine whether miR-193b indeed regulates ErbB4 expression in Ewing Sarcoma, we examined the effects of stable miR-193b expression on ErbB4 protein levels. These analyses revealed robust negative regulation of ErbB4 protein expression by miR-193b in both A673 and SK-ES-1 cells (Fig 4). The proximal ErbB4 3’UTR contains a highly conserved miR-193b predicted binding site (Fig 5A). To determine if this site is responsive to regulation by miR-193b in Ewing Sarcoma cells, we examined its activity in a 3’UTR reporter system. As shown in Fig 5B, in both A673 and SK-ES-1 cells, the wild-type ErbB4 3’UTR site, but not the mutated site, caused robust downregulation of reporter activity in the presence of miR-193b, but not the non-targeting negative miR control. Thus, miR-193b likely regulates ErbB4 expression levels through direct action on the proximal 3’UTR.

### ErbB4 depletion is growth inhibitory in Ewing Sarcoma

**Fig 6:**
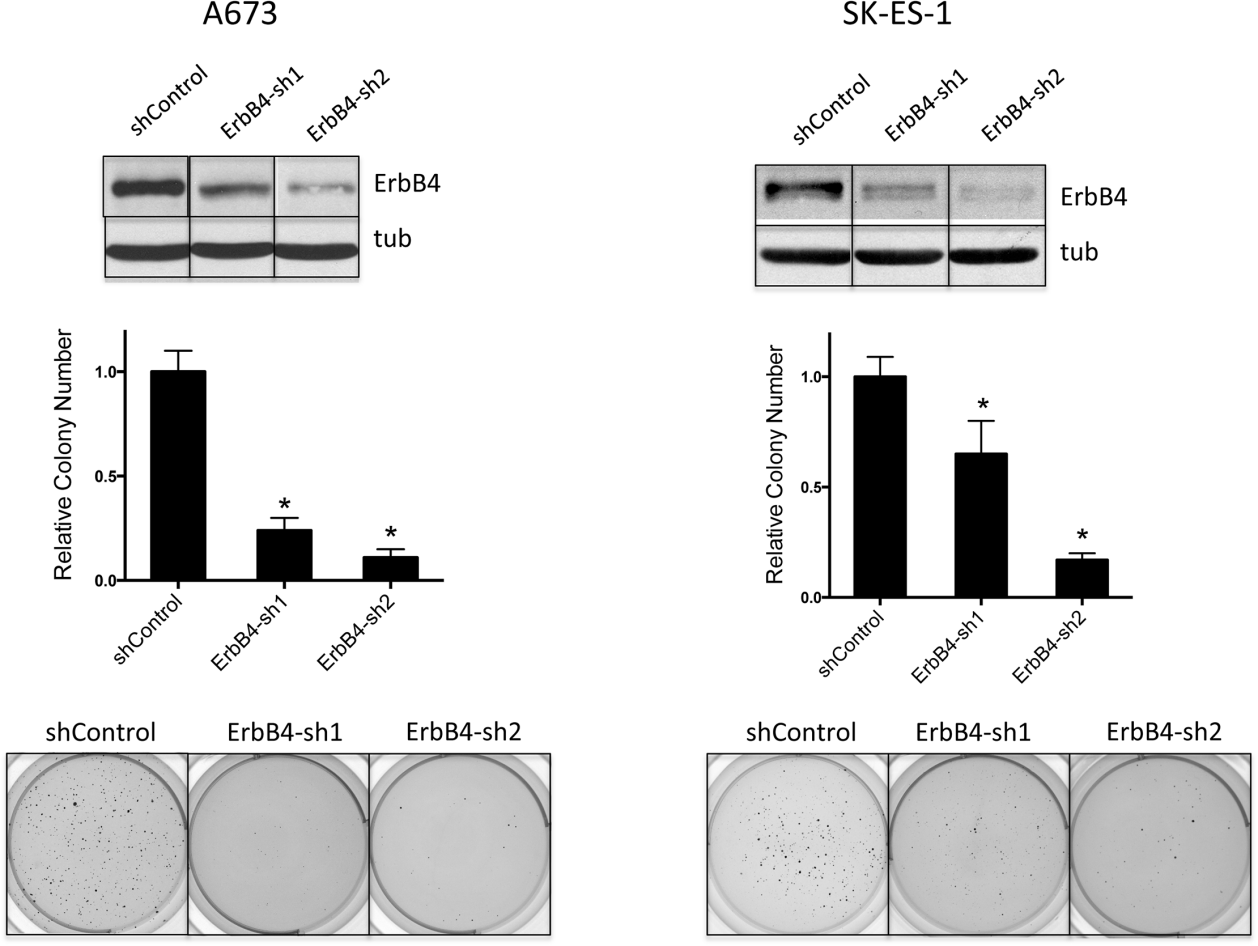
Depletion of ErbB4 results in inhibition of anchorage-independent growth in soft agar. ErbB4 was stably depleted using 2 different shRNAs in A673 and SK-ES-1 cells, as confimed by immunoblotting with ErbB4 antibody and tubulin as loading control. Compared to scrambled shRNA control, ErbB4 knock-down resulted in diminished colony formation with both shRNAs, in both cell lines. Graphs show mean and standard error of the mean of two to three independent experiments, each performed in triplicate; colony counts are normalized to the control, which is arbitrarily set to 1; *p<0.05 using the student t-test. Images from one representative experiment are also shown.

To determine whether downregulation of ErbB4 levels contributes to the growth suppressive phenotype of miR-193b, we stably depleted ErbB4 in A673 and SK-ES-1 cells using lentivirally delivered shRNAs. Compared to control cells (transduced with non-targeting scrambled control shRNA), stable depletion of ErbB4 with two different targeting shRNAs inhibited anchorage-independent growth in soft agar in both cell lines (Fig 6). Thus, negative regulation of ErbB4 expression likely contributes to growth inhibition by miR-193b in Ewing Sarcoma.

## Discussion

Our studies identify miR-193b as a new growth-suppressive microRNA in Ewing Sarcoma, and provide evidence in support of ErbB4 as a mediator of this growth inhibition. As shown in our previous [14] and current work, miR-193b is downregulated in Ewing Sarcoma, at least in part due to action of the EWS/Fli1 oncoprotein, and thus represents a miR amenable to potential therapeutic replacement.

MiR-193b has been identified as a modulator of cancer phenotypes in a number of other contexts. In the vast majority of published literature, miR-193b is reported to be downregulated and to elicit anti-cancer action upon ectopic expression, including inhibition of cell/tumor growth and metastasis. Such action as a tumor/metastasis suppressor miR has been shown in a number of adult epithelial malignancies, including cancers of the breast [21], prostate [22], ovary [23], pancreas [24] and liver [25], and in melanoma [26]. Recurrently identified miR-193b targets relevant to its tumor/metastasis suppressive roles in these contexts have included cyclin D1 [25, 26], and urokinase-type plasminogen activator [21–23]. Tumor suppressive action for miR-193b has also been shown in acute leukemia, both lymphoid [27] and myeloid [28] types. Moreover, in a mouse genetic deletion model, miR-193b has recently been implicated in the restriction of hematopoietic stem cell (HSC) self-renewal [29]. Interestingly, in head and neck squamous cell carcinoma (HNSCC), miR-193b appears to function as a tumor promoter via negative regulation of the NF1 tumor suppressor [30], highlighting the pleiotropic action, and context dependence, of microRNAs.

Our studies identify ErbB4 as a relevant miR-193b target in Ewing Sarcoma. Previous work of others has uncovered an important role for ErbB4 in suppression of anoikis [20], a phenotype of potential relevance to our soft agar assay findings. Interestingly, other studies have also identified ErbB4 as a metastasis promoter in Ewing Sarcoma [31]. As miR-193b has been implicated in metastasis suppression in a number of other cancers [21–23], it will be of interest in future studies to examine the potential role of the miR-193b/ErbB4 axis in Ewing Sarcoma metastasis. Further highlighting the importance of this axis in cancer, two recent studies have reported both tumor and metastasis suppressive functions for the miR-193b paralog miR-193a in lung cancer, through downregulation of ErbB4 [32, 33].

In closing, our studies identify miR-193b as a new growth-suppressive miR in Ewing Sarcoma, and ErbB4 as a relevant candidate target potentially contributing to its growth-suppressive effects. Both molecules are tractable pharmacologic targets, and their therapeutic manipulation could be of interest in Ewing Sarcoma and other cancers.
